# Spatio-Temporal Dynamic Analysis of Sustainable Development in China Based on the Footprint Family

**DOI:** 10.3390/ijerph15020246

**Published:** 2018-02-01

**Authors:** Jing Zhao, Caihong Ma, Xiangui Zhao, Xiaoyu Wang

**Affiliations:** 1 Division of Student Affairs, Xi’an Shiyou University, Xi’an 710065, China; zhjzhj200318@163.com; 2 School of Resource and Environment, Ningxia University, Yinchuan 750021, China; 3 School of Geography and Tourism, Shaanxi Normal University, Xi’an 710062, China; zhaoxg@snnu.edu.cn (X.Z.); babycecilia@126.com (X.W.)

**Keywords:** sustainable development, Footprint Family, ecological footprint, carbon footprint, water footprint, China

## Abstract

The existing index systems on sustainable evaluation are mostly based on a multi index comprehensive evaluation method. The main disadvantage of this approach is that the selection and assignment of evaluation indexes are greatly influenced by subjective factors, which can result in poor comparability of results. By contrast, the Footprint Family (including ecological footprint, carbon footprint, and water footprint) is not affected by subjective factors. The Footprint Family also covers the basic tenets of sustainable development. This paper proposes use of a sustainable development evaluation index system based on the principle of the Footprint Family, and including the ecological pressure index (*EPI*), the ecological occupancy index (*EOI*), the ecological economic coordination index (*EECI*), the GHG (Greenhouse Gas) emission index (*CEI*), the water resources stress index (*WSI*), and the sustainable development index (*SDI*). Furthermore, a standard for grading the evaluated results based on global benchmarks is formulated. The results of an empirical study in China were the following. The development situation deteriorated from 1990 to 2015. The results showed that the *SDI* decreased from a medium level (grade 5) to a lower-medium level (grade 4). The results of this empirical study also showed that the method of evaluation can avoid the influence of subjective factors and can be used in the evaluation of sustainable development for various temporal and spatial conditions.

## 1. Introduction

Since the industrial revolution in the 18th century, the world population has increased from less than 1 billion to more than 7.1 billion. Undoubtedly, the industrial revolution promoted the rapid development of society and the global economy, improving greatly peoples’ living standards. However, a series of global resource and environmental problems have emerged along with considerable social and economic development. Issues such as frequent visibility and haze issues, particularly in cities, as well as climate anomalies, resources exhaustion, and other ecological crises have forced society to revisit the traditional development concept in which economic growth might be a priority if there is a trade-off between environmental sustainability and economic development [1,2,3,4]. In 1987, the United Nations World Commission on Environment and Development (WCED) proposed formally the concept of sustainable development. Since then, the United Nations (UN) and governments around the world all attach great importance to sustainable development. From 1992 to 2002, the UN held three Sustainable Development Earth Summits. Sustainable development has become a major issue, which arouses general concern in academia and various circles in society. The underpinning theory behind sustainable development is that biodiversity, ecosystem processes, and ecosystem services need to continue; the economy, society and human well-being need to develop; economic development and environmental protection are not completely contradictory but complement each other [5]. Sustainable development ought to be safeguarding long-term ecological sustainability, satisfying basic human needs and promoting intergenerational equity [2].

How do we evaluate sustainability? This is a vital issue which must be addressed if we are to progress research on sustainable development [6]. In 1992, at the first Sustainable Development Earth Summit in Rio de Janeiro, many scientists had proposed the need to establish a sustainable development assessment methodology. Since then, some international organizations, government agencies, academic groups, and many scientists have actively promoted research and application of sustainable development assessments; consequently, a variety of assessment methods and index systems have been reported [7]. However, the different methods and index systems have their own unique advantages and disadvantages. Up to now, a satisfactory and universally accepted sustainable development evaluation system has not been established. The United Nations Development Program (UNDP) adopted a Human Development Index (*HDI*) as an evaluation index, and has published several annual Human Development Reports since 1990. At the Rio + 20 summit in 2012, UNDP proposed that the *HDI* should be replaced by the Sustainable Development Index (*SDI*) to measure social development in a more comprehensive way. However, the calculation method for the *SDI* requires further investigation [1]. It is worth mentioning that China has done a lot on the assessment of sustainable development [8,9]. For example, a Green Development Index evaluation system was proposed in 2010 and the Chinese Green Development Index Report (CGDIR) was published for 4 consecutive years by scholars in Beijing Normal University. In addition, a Sustainable Development Index System (SDIS) based on hierarchical analysis was proposed by the sustainable development strategy team of the Chinese Academy of Sciences [4]. In conclusion, there has been great progress in research for the quantitative evaluation of sustainable development, but there are still some shortcomings. The main drawback of the existing multi-index comprehensive evaluation methods is that the selection and assignment of evaluation indexes are greatly influenced by subjective factors. This major deficiency leads to a lack of comparability between different sustainability methodologies.

In 1997, an ecological footprint (*EF*) method was proposed [10]. Compared with the numerous multi-index integrated sustainable development evaluation index systems, the *EF* analysis method represents a breakthrough in evaluation methods of sustainable development. Since the inception of the *EF* method, it has been widely used in the evaluation of sustainable development [8,11,12]. Based on the idea of an *EF*, several environmental footprints such as energy footprint [13], water footprint (*WF*) [14], carbon footprint (*CF*) [15], nitrogen footprint (*NF*) [16], biodiversity footprint (*BF*) [17], and economic footprint [11,18] have been proposed in succession. To assess the comprehensive impact of human beings’ activities on the planet, Galli et al. [19,20] proposed a comprehensive footprint named the Footprint Family. The Footprint Family is a collection of many footprints. For the reason that many footprints lack clear accounting methods, except in the case of the *EF*, the *CF,* and the *WF*, the Footprint Family is often regarded as consisting of the *EF*, the *CF,* and the *WF*. So, the Footprint Family can usually be adopted for the evaluation of human consumption of biological resources, the impact on the environment of water resources utilization, and GHG emissions.

Different from the multi-index comprehensive evaluation method, the Footprint Family method can avoid the influence of subjective factors. This method can objectively evaluate the ecological supply and demand, greenhouse gas (GHG) emissions, and water resource utilization. Therefore, the Footprint Family concept occupies an important role in the quantitative assessment of international sustainable development. For example, the European Union (EU) has funded a project named One Planet Economy Network (OPEN) to monitor sustainability of the European economy, because the European Commission (EC) considered that the environmental impact of the European economy was nearly three times what the planet can cope with. Additionally, the evaluation method for the project is based only on the Footprint Family concept [11,19,20,21].

Compared with the traditional multi-index comprehensive evaluation method, the Footprint Family method takes sustainable development evaluation to a new level, but the method still has some shortcomings. First, it lacks indicators that can reflect the level of regional economic development. Second, it is not easy to refine or incorporate relevant indicators into the Footprint Family. No specific guidance on these points is given in the current Footprint Family method. Third, it lacks criteria on how best to grade the evaluated results. In this paper, the authors have improved on the Footprint Family method, put forward some new evaluation indexes, built a new evaluation index system of sustainable development from a new perspective, and formulated the grading criteria for the evaluated results. Sustainable development in China was evaluated at two levels: the national and the provincial scale, including analysis of both the spatial and temporal variation characteristics. The results provide a new method for sustainable development evaluation, in addition to providing a robust decision-making reference for China’s sustainable development.

## 2. Methods

### 2.1. Study Area

**Figure 1:**
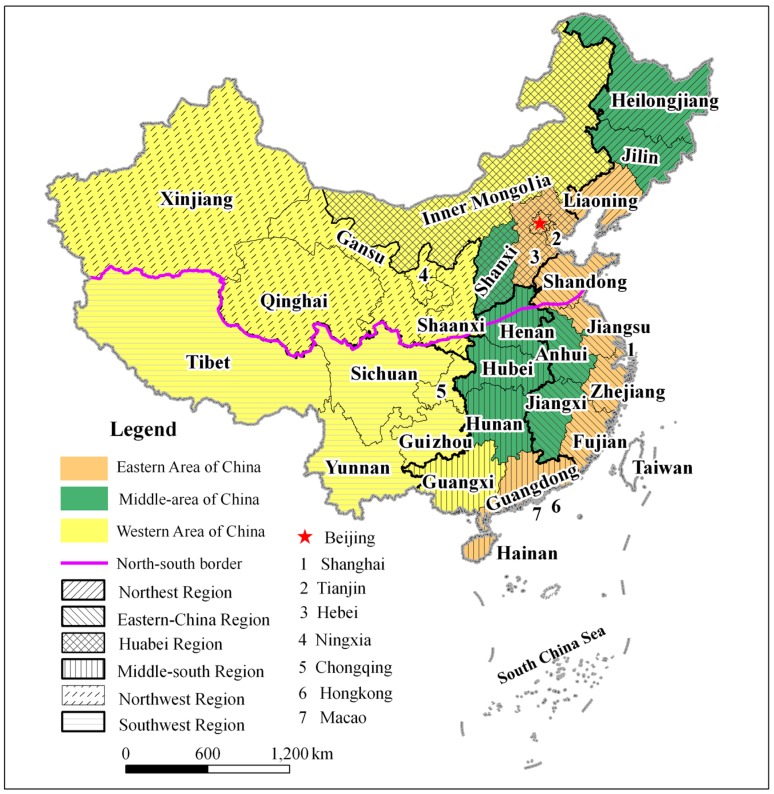
Map of the study area.

The study area includes 22 provinces, 5 autonomous regions, and 4 municipalities directly under the control of the central government of China, viz., the 31 provincial administrative districts (PADs). Due to a lack of data, Hong Kong, Macau, and Taiwan were not included in the study. Traditionally, China has been divided into three parts, viz., the eastern region, the central region, and the western region. According to the administrative and geographic location, the study area was divided into six parts, that is, the Northeast region, the Eastern region, the Huabei region, the Middle-south region, the Northwest region, and the Southwest region. In addition, China can be divided into a northern and a southern region by a north-south border, which extends along the Qinling Mountains and the Huaihe River (Figure 1).

### 2.2. The Conceptand Calculation Methodof the Footprint Family

#### 2.2.1. Ecological Footprint

The *EF* method includes three important indicators, i.e., the *EF*, the ecological capacity (*EC*), and the ecological deficit (*ED*). According to the definition of the Global Footprint Network (GFN) [22]. The *EF* refers to a measure of the requirements of human activities in the biosphere under current technology and the resource management level. The *EF* can be divided into two types, i.e., a renewable type and a non-renewable type, termed the biomass ecological footprint (*BEF*) and the energy ecological footprint (*EEF*), respectively. The *EC*, sometimes referred to as biocapacity by some scholars, refers to a measure of the amount of biologically productive land and sea area available to provide the ecosystem services that humanity consumes. The surplus value of *EF* minus *EC*, termed the *ED*, can be divided into two types, the hard *ED* (*HED*) and the soft *ED* (*SED*), in which *HED* refers to the biomass deficit and *SED* means there is an ecological deficit but a biomass surplus [23]. The formula for calculating the *EF* is as follows:

$$EF=N\times ef=N\sum_{i=1}^{n}\left(a_{i}\times r_{j}\right)=N\left[\sum_{i=1}^{n}\left(c_{i}/p_{i}\right)\right]\times r_{j},\;(i=1,\;2,\;3,\;\ldots ,n;j=1,\;2,\;3,\;4,\;5,\;6)$$

$$EC=N\times ec=N\sum_{j=1}^{6}\left(a_{j}\times r_{j}\times y_{j}\right),\;(j=1,\;2,\;3,\;\ldots ,\;6)$$

in which *EF* is the regional ecological footprint (hm^2^), *EC* is the biocapacity (hm^2^), *N* is the population, *ef* is the ecological footprint per capita, *ec* is the bio-capacity per capita, *a_i_* is the amount of biologically productive land per capita of substance *I*, *r_j_* is the equivalence factor, *c_i_* is the consumption per capita of substance *I*, *p_i_* is the average world production capacity for the substance, *i* is a type of consumptive substance, *j* refers to the amount of biologically productive land, *a_j_* is the actual amount of biologically productive land per capita, and *y_j_* is the yield factor.

This paper adopts the equivalence factors of GFN: The equivalence factor of arable and building land is 2.51, the water area is 0.37, the grassland is 0.46, and both the woodland and carbon absorbed land is 1.26. The yield factor (*YF*) of cropland and built-up land is the ratio of the total grain output of China to the global total grain output per year. Additionally, the *YF* of the rest of the land types is the average value that has been adopted by different scholars for the corresponding land use type [24,25,26]. So, the *YF* of grassland is 0.34, forest land is 0.91, and water area is 0.81.

#### 2.2.2. Carbon Footprint

The *CF* measures the direct and indirect GHG emissions from human activities [19,20]. This paper adopts the method recommended by the Intergovernmental Panel on Climate Change (IPCC) 2006 Guidelines for National Greenhouse Gases Inventories and the Chinese Guidelines for Provincial Greenhouse Gases Inventory based on IPCC, and the calculation formulas and factors obtained from the literature [27,28]. The unit of *CF* is t CO_2_e, which is an equivalent of CO_2_, that is, a term for describing different greenhouse gases in a common unit. The value of *CF* is the sum of each kind of GHGs emissions multiplied its corresponding GHG global warming potential (GWP).

#### 2.2.3. Water Footprint

The *WF* can be divided into the direct water footprint (*DWF*) and indirect water footprint (*IWF*). The *DWF* refers to the consumption of existing water, whereas *IWF* refers to the consumption of virtual water [29,30]. The formula for calculating the *WF* is as follows:

$$WF=WU+\sum_{i=1}^{n}(P_{i}\times VWF_{i}),\;(i=1,\;2,\;3,\;\ldots ,\;n)$$

in which *WF* is the regional water footprint (m^3^), *WU* is the entity water usage, *P_i_* is the consumption of eventual consumer goods *I*, and *VWF_i_* is the virtual water content per unit product of *i*, whose value can be found in the literature [31,32].

#### 2.2.4. Data Sources

Yield, consumption, and import and export data used in this study were obtained from the China Statistical Yearbooks, the China Energy Statistics Yearbooks, the China Agriculture Statistics Yearbook, the statistical yearbooks of each province-level administration, the Chinese Economic and Social Development Statistical Database, the UN Food and Agriculture Organization Statistics Database, the New China 50 Years Statistical Data Collection, and the National Statistical Yearbook of China. In addition, some data were obtained from the websites and relevant databases of Chinese governmental agencies, such as the National Bureau of Statistics, State Forestry Administration, and the Ministry of Land and Resources.

### 2.3. The Construction of the Sustainable Development Evaluation System

Based on the concept of the Footprint Family, this paper proposes a sustainable development index (*SDI*) as a measure of sustainability. It is a composite index comprising the ecological pressure index (*EPI*), the ecological occupancy index (*EOI*), the eco-economic coordination index (*EECI*), the GHG emission index (*CEI*), and the water resources stress index (*WSI*). In this paper, each index of the sustainable development evaluation system was standardized by the extreme method. The maximum values of the *EPI*, *EOI*, *EEC,* and *WSI* were 2, 3, 4 and 1, respectively.

The *EPI* is the ratio of *BEF* to *EC* for a given region. This can reflect the degree of regional ecological environment pressure.

The *EOI* is the ratio of *ef* of a given region to the global *ef* in the same period. The *EOI* can reflect the regional social and economic development and consumption level.

The *EECI* is the ratio of *EOI* to *EPI*. This can reflect the coordination between socio-economic development and the ecological environment.

The *CEI* is synthesized from the per capita GHG emission index (*C_p_*) and the per unit GHG emission index (*C_a_*). The *CEI* can evaluate the contribution of regional GHG emissions to global climate change. The formula for calculating *CEI* is as follows:

$$CEI=W_{p}\times \frac{C_{p}}{C_{p.\max}}+W_{a}\times \frac{C_{a}}{C_{a.\max}}$$

in which *C_p_* is the ratio of regional carbon footprint per capita to the value that was set as the global climate change mitigation goal, and *C_a_* is the ratio of carbon footprint density (*CFD*) of a given region to the value that is set as the global climate change mitigation goal. The Stern Report puts forward the global per capita limitas 2 t CO_2_e as a national goal to undertake the emission reduction obligations [33]. In 2008, the global bio-capacity was 119 × 10^8^ hm^2^ [34] as announced by WWF, so the *CF* density coupled with global climate change targets was 1.68 t/hm^2^. *C_p._*_max_ is the maximum value of global *CF* per capita, which is 15. *C_a_*_.max_ is the maximum value of *CF* density, which is 20. *W_p_* and *W_a_* are the weights of *C_p_* and *C_a_*, respectively. In the present paper, 32 experts in the field were selected for the questionnaire survey, and 32 copies were returned and validated. The weight values given by the experts were processed by a weighted average method, and the values of *W_p_* and *W_a_* were 0.6 and 0.4, respectively.

The *WSI* is the ratio of regional *WF* to the amount of water resource available. The *WSI* can reflect the degree of water resources pressure.

The *SDI* is used to evaluate the overall condition of regional sustainable development. It is assumed that the five indices above have equal weight in the evaluation of sustainable development, and differentiate positive and negative indicators in the calculation of *SDI*. The formula for calculation of the *SDI* (0 ≤ *SDI* ≤ 1) is as follows:

SDI=1/5[(1−EPI)+EOI+EECI+(1−CEI)+(1−WSI)]

The classification standard of the corresponding index is shown in Table 1.

## 3. Results and Analysis

### 3.1. Spatio-Temporal Dynamic Analysis of EF

#### 3.1.1. Dynamic Changes of *ef* (1990~2015)

**Figure 2:**
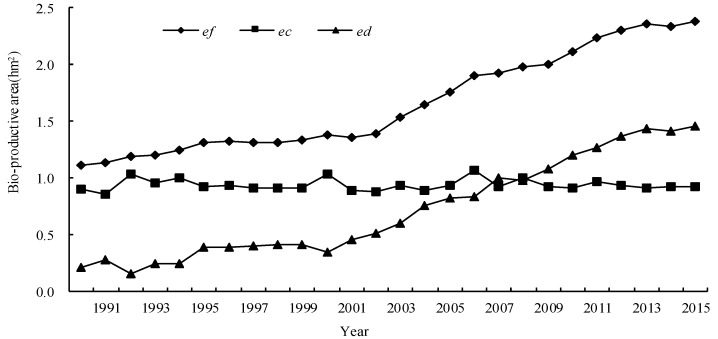
Dynamic changes of the ecological footprint and biocapacity per capita. *ef* is the ecological footprint per capita; *ec* is the biocapacity per capita; *ed* is the ecological deficit per capita.

During the research period, the *ef* increased from 1.11 hm^2^ to 2.38 hm^2^ in 2015 with an average annual growth rate of 3.09%, while the *ec* fluctuated between 0.86 and 1.07 hm^2^; the *ed* increased from 0.21 hm^2^ to 1.45 hm^2^ with an average annual growth rate of 8.11% (Figure 2).

The above figure indicates that the consumption of natural resources has increased year by year, and has exceeded the carrying capacity of the ecosystem. The yield factor of cropland increased 28.81% since 1990 on account of the development and application of agricultural science and technology, so the biocapacity has improved much. However, because cropland has decreased by 6.38% and the population has increased by 19.88%, the increase of biocapacity per capita was partially offset by these factors. Additionally, this partial deficiency in biocapacity was almost made up for by a favorable surplus in the export trade volume over these years in China.

Over the past 25 years, comparing 2015 with 1990, the *ef* has increased as follows: the *ef* of fossil fuel land has increased 3.00 times; water area, 2.95 times; grassland, 2.47 times; forest land, 1.62 times; cropland, 1.38 times; and built-up land, 1.29 times. These data showed that the maximum increase in consumption per capita was associated with energy, aquatic products, and animal products, whereas grain consumption per capita increased little since 1990. In addition, major changes have taken place in the structure of Chinese food consumption and consumption of high nutrition, and high calorie food shaving greatly increased since China adopted a reform and opening-up policy in 1978.

In 2015, the value of 6 components of *ef* decreased in the following order: fossil fuel land (52.54%) > cropland (29.58%) > water area (5.86%) > built-up land (5.29%) > grassland (4.05%) > forest land (2.69%). Chinese energy consumption was dominated by coal, whereas biological resources consumption was dominated by agricultural products. This excessive dependence on non-renewable and natural resources is the main cause of *ed* in China. An energy footprint accounted for more than 50% of the total *ef*, so a good way of reducing coal and oil consumption is by developing new energy sources such as biomass energy power generation, given that China is endowed with a strong agricultural sector. It would be realistic to significantly reduce the *ef* and reduce pollution due to biomass burning by making full use of biomass generation.

#### 3.1.2. Spatial Pattern of *ef*

**Figure 3:**
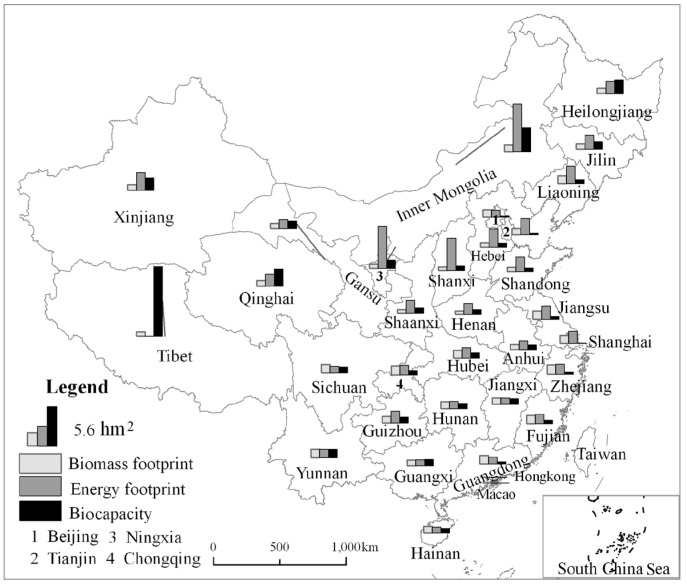
Spatial pattern of the ecological footprint and biocapacity per capita.

At the spatial level, the *ef* varied widely in 2015. The *ef* was very high in the provincial administrative districts (PADs) such as Inner Mongolia (8.86 hm^2^), Ningxia (7.57 hm^2^), and Shanxi (5.81 hm^2^), and the *ef* values were very low in the PAD of Tibet (0.7 hm^2^), Guangxi (1.88 hm^2^), and Jiangxi (2.00 hm^2^). This distribution of *ef* was mainly due to the component of the energy footprint, which is at a high percent level in the northern regions (Figure 3). The *ec* was very high in the PAD of Tibet (11.26 hm^2^), Inner Mongolia (3.88 hm^2^), and Qinghai (2.74 hm^2^), but in some PADs it was very low, for instance, it was only 0.08 hm^2^ in Shanghai, 0.16 hm^2^ in Beijing, and 0.20 hm^2^ in Tianjin. The majority of PAD *s* were in the state of *ed* except Tibet.

The main reason for the above variations in *ef* was connected to the distribution of fossil energy consumption and heavy industry. In winter in the northern regions, there is a need for high energy consumption for heating due to the extreme cold. Besides, more heavy industries with high fossil energy consumption are located in the northern region, which leads to a higher *CF* in the northern region compared to the southern region. Thus, the key to reducing the *CF* is to adjust the industrial structure and distribution, improve energy efficiency, reduce energy consumption, and develop a green economy.

### 3.2. Spatio-Temporal Dynamic Analysis of CF

#### 3.2.1. Dynamic Changes of *CF* (1990~2015)

**Figure 4:**
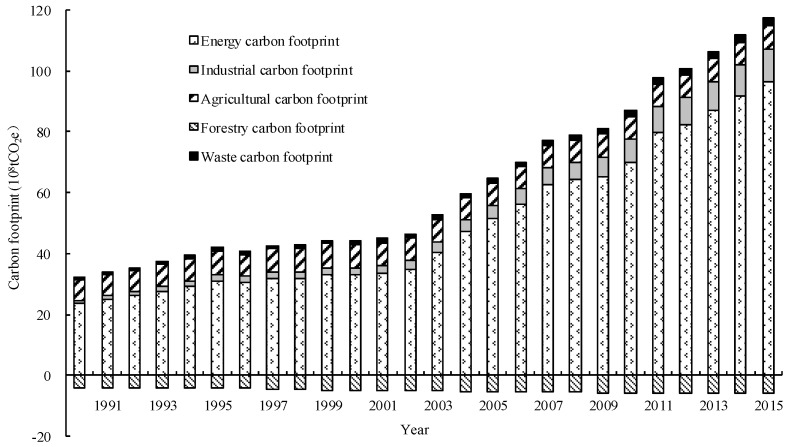
The component and dynamic changes of the carbon footprint.

The *CF* in China increased rapidly from 1990 to 2015 (Figure 4). The *CF* increased from 36.23 × 10^8^ t to 123.32 × 10^8^ t with an average annual growth rate of 5.02%. In this, the energy *CF* increased from 23.60 × 10^8^ t to 96.62 × 10^8^ t, the industrial *CF* increased from 0.85 × 10^8^ t to 10.71 × 10^8^ t, the agricultural *CF* increased from 6.80 × 10^8^ t to 7.32 × 10^8^ t, the waste disposal *CF* increased from 0.79 × 10^8^ t to 2.63 × 10^8^ t, and the forestry carbon sequestration increased from 4.20 × 10^8^ t to 6.04 × 10^8^ t.

Regarding the components of the *CF*, in 2015 the percentage of each type of *CF* was as follows: energy (82.38%), special industry (9.14%), agriculture (6.24%) and waste disposal (2.28%); and forestry carbon sequestration can neutralize 5.4% of the total CF of the year. This showed that the *CF* in China was mainly caused by fossil fuel combustion, and also that forestry carbon sequestration has great potential for amelioration. The *CF* of industrial production increased at an average annual rate of 11.3%, and it accounted for 5.84% of fossil energy consumption, 5.05% of waste disposal, 1.45% of forestry carbon sequestration, and 0.35% of agriculture. Clearly, the maximum increase of *CF* was due to industrial production, which was directly related to the rapid development of infrastructure construction and the sharp increase in demand for and the output of cement during the 25 year period.

#### 3.2.2. Spatial Pattern of Carbon Footprint

**Figure 5:**
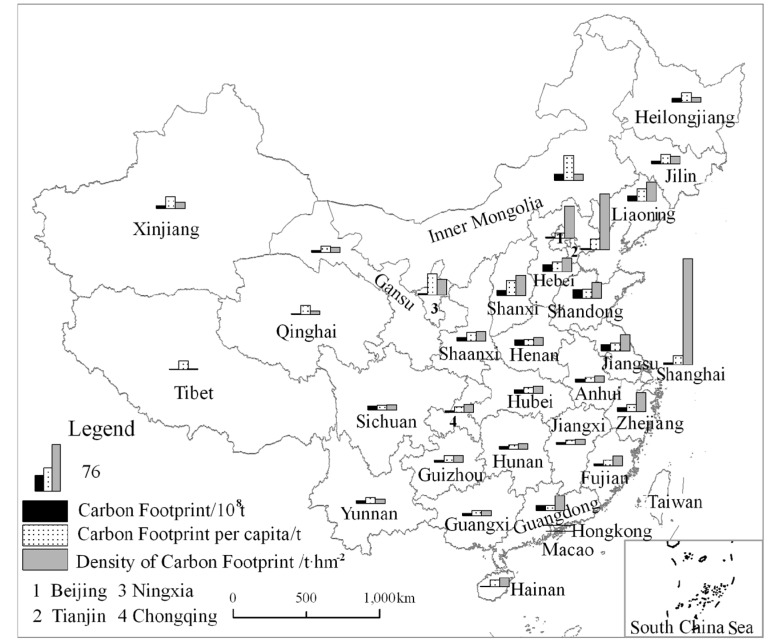
Spatial pattern of the carbon footprint

At the spatial level, the *CF* in the eastern region was higher than other regions of China. For instance, the values of the *CF* in Shandong, Hebei, and Jiangsu were among the very highest of the 31 PADs of China. The *CF* per capita of each PAD was higher in the northern region than the southern region, and the *CFs* were very high in Inner Mongolia and Ningxia. The *CF* density has a spatial distribution feature that gradually reduced going from east to west (Figure 5).

China’s traditional economy is very dependent on fossil energy and the country remains a high-carbon economy. In recent years, China has championed the green and low-carbon economy in response to global climate change requirements. In general, economic development in the eastern region is higher than in the central and western regions. Therefore, the spatial differences in *CF* reflect, to a considerable extent, the spatial imbalances of economic development. Overall, the level of China’s economic development tends to decrease going from east to west. So, the regional *CF* and *CF* densities have been higher numerically in the eastern region. Another reason for such differences is connected with the structure of energy consumption. Many provinces in the northern region have abundant fossil fuel resources such as coal, petroleum, and natural gas. This then leads to most heavy industry factories being located there, hence causing a high concentration of secondary industries in the northern region. In addition, the high weight of coal consumption almost dominates the structure of energy consumption in the northern region. While consuming the same amount of coal and natural gas, coal discharges amount to 50% more CO_2_e emissions than those of gas.

### 3.3. Spatio-Temporal Dynamic Analysis of WF

#### 3.3.1. Dynamic Changes of *WF* (1990~2015)

**Figure 6:**
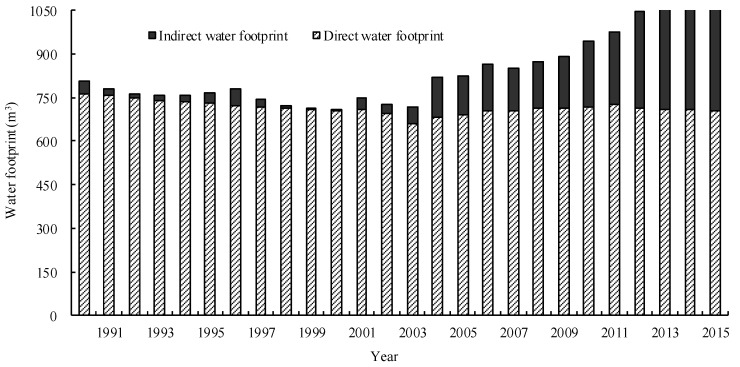
Dynamic changes of the water footprint.

The *WF* per capita increased from 805.10 m^3^ to 1079.31 m^3^, with an average annual rate of 1.18% (Figure 6). The entity water consumption per capita decreased from 764.00 m^3^ to 704.61 m^3^, with an average annual rate of −0.32%; virtual water imports per capita increased from 41.04 m^3^ to 374.70 m^3^, with an average annual rate of 10.01%. The *WSI* increased from 0.37 to 0.53. This showed that the *WSI* in China is relatively low on a country scale basis. Since 1990, the decreased entity water consumption and the sharply increased virtual water importation were commensurate with a large number of water imports, which was conducive to the protection of Chinese water resources.

#### 3.3.2. Spatial Pattern of *WF*

**Figure 7:**
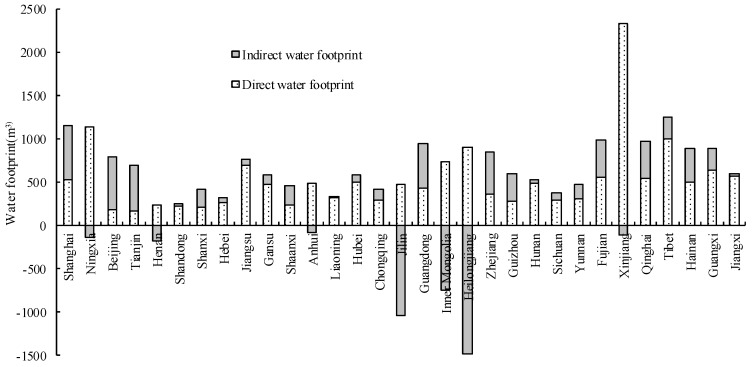
Spatial pattern of the water footprint per capita.

The *WF* per capita administration data were very different at a spatial level. In 2015, the WF values were high in Xinjiang (2210.71 m^3^), Tibet (1243.94 m^3^), and Shanghai (1153.33 m^3^), while values were very low in Heilongjiang (−583.35 m^3^), Jilin (−564.01 m^3^), and Inner Mongolia (−15.30 m^3^). A negative value of *WF* per capita means the virtual water exports exceeded the entity water consumption. The maximum entity water consumption values were for Xinjiang (2325.60 m^3^), Ningxia (1130.36 m^3^), and Tibet (1001.47 m^3^), while the minimum values were for Tianjin (167.23 m^3^), Beijing (174.79 m^3^), and Shanxi (202.61 m^3^). The maximum virtual water imports were for Shanghai (632.74 m^3^), Beijing (619.52 m^3^), and Tianjin (527.32 m^3^), and the maximum exports were for Heilongjiang (1485.31 m^3^), Jilin (1032.54 m^3^), and Inner Mongolia (745.60 m^3^) (Figure 7).

The spatial difference in *WF* per capita was mainly affected by factors such as the amount of available water resources, climate, industry and agricultural production, population density, and living habits. In 2015, the amount of available water resources per capita in Beijing, Tianjin, Henan, Hebei, Shandong, Shanxi, and other provinces was low (<350 m^3^); the entity water consumption and the *WF* per capita in these provinces were also low (the former < 300 m^3^, the latter < 700 m^3^). However, the entity water consumption and the *WF* per capita in Shanghai, whose amount of available water resources per capita is the lowest in the country (<150 m^3^), exceeded 500 m^3^ and 1000 m^3^, respectively. This is possibly related to the high population density and the high consumption living habits of the Shanghai population. The entity water consumption and the amount of available water resources per capita in Xinjiang reached 2325.6 m^3^ and 4055.5 m^3^, respectively. Overall, the water stress is not high, and in a partial drought, water shortages are inevitable. In Heilongjiang and Jilin, the amount of available water resources per capita are in the medium level category; the entity water consumption was 700~900 m^3^, and virtual water exports were caused by the fact that large exports of farm and pasture products exceeded entity water consumption by 2.09~120.38%. In comparison, virtual water imports in Shanghai, Beijing, Tianjin, and Guangdong accounted for 54.86~78.0% of *WF*. This showed that it was possible to reallocate water resources, reduce water stress in water-deficient areas, and utilize water resources more effectively through virtual water imports and exports in addition to the trans-regional distribution of water resources.

### 3.4. Evaluation of Sustainable Development

#### 3.4.1. Dynamic Changes of Sustainable Development (1990~2015)

**Figure 8:**
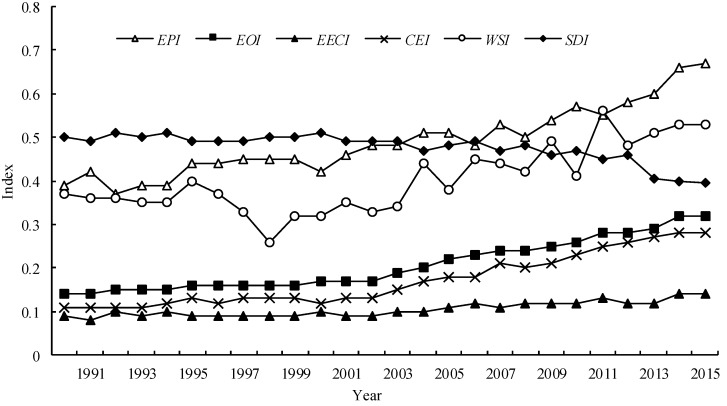
Dynamic changes of the sustainable development index.

From 1990 to 2015, the *SDI* decreased from a medium level (grade 5) to a lower-medium level (grade 4), the *EPI* increased from a very low level (grade 2) to medium level (grade 5), the *EOI* decreased from an extremely low level (grade 1) to a low level (grade 3), the *EECI* maintained an extremely low level (grade 1), the *CEI* increased from a low level (grade 3) to a lower-medium level (grade 4), and the *WSI* increased from a low level (grade 3) to a medium level (grade 5) (Figure 8).

#### 3.4.2. Spatial Pattern of Sustainable Development

**Figure 9:**
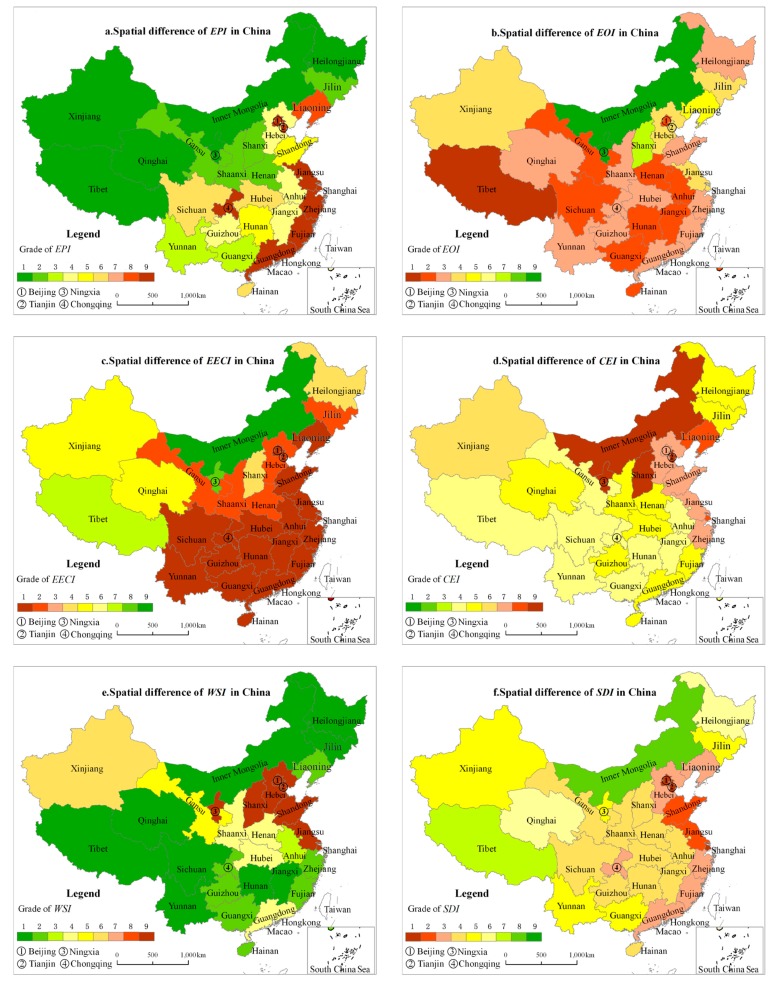
Spatial pattern of the sustainable development index.

In 2015, the *SDI* was high in Inner Mongolia and Tibet, low in the eastern region, and medium in other regions. The *EPI* in Tibet, Inner Mongolia, Qinghai, Heilongjiang, Ningxia, and Xinjiang belonged to the lowest category, while those in Beijing, Tianjin, Shanghai, Jiangsu, Zhejiang, Fujian, Guangdong, and Chongqing were at the highest (Figure 9). The *EOI* was relatively high in Inner Mongolia, Ningxia, and Shanxi; medium in Hebei, Xinjiang, Tianjin, Jiangsu, Shanghai, and Jilin; and low in other provinces. The *EECI* was good in Inner Mongolia, Ningxia, and Tibet; medium in Qinghai, Xinjiang, Shanxi, and Heilongjiang; and poor in other PADs. A higher degree of *CEI* was concentrated in North China and East China, and was medium in other PADs. A higher degree of *WSI* was concentrated in North China and East China, medium in Northwest and Middle-of-south, and relatively low in other PADs.

## 4. Discussion

### 4.1. Improvements on the Footprint Family

Improvements on the Footprint Family are mainly embodied in the following two aspects:

(1). Improvements in the *EF* method. These improvements mainly refer to construction of 3 new indicators—the *EPI*, the *EOI,* and the *EECI*—based on the principle of the *EF*. The current method of *EF*-evaluated sustainable development with *ED* as the index results in the conclusion that in a region with a higher level of social economic development, the *ED* is higher, and sustainability is worse. It seems that economic development and sustainable development are opposing each other. The main reason is that the energy footprint pressure is borne by the consumption area entirely, and this does not accord with the actual situation. For example, the energy footprint per capita in the USA is up to 4.87 hm^2^. But the resulting series of adverse effects not only affect the United States, but also affect other countries and regions because of the atmospheric circulation. That is, the whole world has to bear the energy and environmental problems caused by the high energy consumption. The analysis in this paper shows that the *EPI*, defined by the *EF* of renewable resources, can be used to reflect more accurately the regional ecological pressure caused by human consumption. Additionally, the *CF* can be used to evaluate, more comprehensively and scientifically, the responsibilities of global changes caused by GHG emissions due to energy consumption. This new approach also eliminates the overlap of energy footprint and *CF*. With respect to the problems of *EF* and sustainable development, existing studies indicate that countries should increase their share of global footprint, rather than merely reduce the *EF*, to achieve sustainable development [35]. The *EOI* in this study is a manifestation of this concept. The *EOI* based on the *EF* method, whose method of calculation and parameters are unified globally, is a more scientific economic development index than currency or GDP. In addition, based on the *EPI* and the *EOI*, an index named *EECI* was constructed to reflect the coordination level between social-economic development and ecological environment.
(2). Improvements on the *CF* method and an index of *CEI* was given. The main purpose of research on a regional *CF* is to assess the contribution of regional GHG emissions to global climate change by measuring regional carbon budgets. Because of different populations and areas for the different regions, when different regions are compared, it is not valid to use only the regional total *CF* or *CF* per capita as the index. In the present work, the authors use the *CF* per capita and the *CF* per unit area to eliminate the influence of population and area; we convert the area of a region into an ecologically productive area and thus represent its biocapacity; in this way we increase the comparability between regions; in addition, we use the *CF* per capita and per unit area of the target set for the control of global climate warming as the benchmark to construct a GHG emission index. At the same time, the authors have established an evaluation standard for the degree of GHG emissions based on the whole world, which did show good discrimination in a test evaluation of the world’s 30 highest GHG emission countries and the PADs of China. Based on the improvement of the CF, an index of *CEI* was given in this study.

Moreover, existing study results differ greatly due to the differences of study methods and data sources [36,37]. For example, the *EF* can be calculated by the yield and import and export trade adjustment at the national scale. But within a country, the *EF* can only be calculated by consumption, because of the inability to obtain real trade data between the regions. The so-called productive *EF* calculated with yield [35] is also inconsistent with the *EF* method. In this paper, the authors appeal that the *EF* should be calculated by the latest methods recommended by The Global Footprint Network [22] as much as possible, and the regional *CF* should be based on the IPCC method. The improvement of internationally recognized methods should be cautious. Generally, it is not needed to change the calculation method and framework at will, so as to ensure that the calculation methods adopted are in line with international research and make the research results more comparable.

### 4.2. Construction of Sustainable Development Evaluation System

The sustainable development theory considers that poverty represents the worst category of unsustainable development [1,35]. Sustainable development is a new development model designed to protect the ecological environment by control of ecological environment capacity and resource carrying capacity. Therefore, sustainable development evaluation should be taken into consideration along with ecological pressure, economic development level, eco-economic coordination, environmental protection, resource constraint, and other aspects. In this paper, the sustainable development index based on the Footprint Family can evaluate comprehensively the regional sustainable development from 5 aspects—ecological pressure, economic development, eco-economic coordination, climate change, and resource constraint. This evaluation index system is a promising-prospect evaluation system, which accords with the aims of ecological civilization construction and human society sustainable development, helps to promote harmonious unification of the economy, society, and ecology, constructs a harmonious society, and achieves sustainable development.

### 4.3. Suggestions for Reducing the Environmental Burden

The areas with high ecological pressure, including Beijing, Tianjin, Shanghai, and Chongqing, are characterized by a large number of people and a sharp contradiction between people and land. The way to reduce the ecological pressure is to keep the ecological red line of cultivated land strictly and prevent the loss of land resources. We should promote the high and new technology of agriculture, give full play to the potential of existing resources, and improve the rate of land output and land carrying capacity. The second way is to develop the ecotourism industry vigorously. We should make full use of local tourism resources and vigorously develop the ecological tourism industry.

The theory of sustainable development holds that poverty is the most unsustainable, and sustainable development requires development as the first priority. For areas with low *EF*, such as Tibet, Guangxi, Jiangxi, Hainan, and Anhui, economic development should be developed to promote poverty alleviation and improve living standards. However, for areas with high ecological occupancy, a conservation-oriented social consumption system should be advocated to change the production and consumption patterns of people. Under limited resources, the biocapacity is also limited, and excessive pursuit of enjoyment of life results in an increase in the *EF*. For example, the *EF* per capita of the United States is as high as 10.9 hm^2^, which is the highest in the world (Table 2), while the global biocapacity per capita is only 1.7 hm^2^. If people all over the world consume according to the American Standard, we would need 6.41 earths to accommodate the consumption. However, human beings have only one earth, and the consciousness of the “community of human destiny” advocated by China is the embodiment of this concept of sustainable development.

For areas with high GHG emission pressure, such as areas in the North China Region and the East China Region, one main path being followed is the transition from a traditional economy to a low-carbon economy, and this process should be accelerated. Alternative approaches can be as follows: (1) Government should raise the price of coal appropriately according to economic conditions to guide residents towards use of green and low-carbon consumption by the means of market regulation. (2) New energies such as natural gas, solar energy, and wind energy should be promoted to optimize the structure of energy consumption and reduce the consumption of high-carbon energy sources. (3) Establish new industrial parks and industrial networks according to the circular economic model, in addition to accelerating the transformation of high carbon emission enterprises, and actively promote the upgrading of low-carbon structures in industry. (4) Implement a green performance appraisal system, and link the completion of energy-saving emission reduction targets at all levels with leadership performance and promotion. Another important path in areas of high GHG emission pressure is to develop carbon trading markets to regulate carbon emissions through the market economy. The opening up of the carbon trading market in China in 2017 should be accelerated to establish and improve gradually the local carbon trading market conditions.

For the areas with high water resource demand, such as in North China, East China, and Northwest China, there are the two main pathways for solving the dilemma. One way is to try to enlarge the available water resources, while the other way is water conservation. The main ways of enlarging the available water resources include enhancing the utilization rate of river water resources by building reservoirs, improving rainwater harvesting via increased use of cellars and ponds, and advancing sewage treatment processes to realize sewage as a renewable resource. With respect to water saving, the main opportunities relate to water conservation in agricultural activities, industrial production processes, and water consumption in daily life. Water conservation in agricultural production activities can be realized by optimizing the planting regime such as reducing the area of rice planting, and using water saving technologies such as drip irrigation, sprinkler irrigation, seepage irrigation, and mulch covering, etc. Industrial water saving measures generally rely on water saving technology transformation for enterprises with large water consumption and using obsolete technologies, to achieve an increased reuse rate for industrial water of more than 70%. With respect to government actions, on the one hand, guidance should be given to residents on practical water saving measures and the real costs of producing clean water, while at the manufacturing and engineering level, there is a need to introduce and modernize water-saving facilities and infrastructure. For instance, according to the requirements for a water-saving society, with respect to market requirements for sanitary ware, sales of units that are designed to consume water of more than 9 L/single flush should be prohibited and instead water-saving flush toilets consuming less than 6 L/single flush should be advocated.

### 4.4. Division of Index Grade Evaluation and the Results of Global Assessments

Based on the footprint family, the *EPI*, the *EOI*, the *EECI*, the *CEI*, the *WSI,* and the *SDI* taken together are all proposed as an evaluation system, whereby evaluation factors and calculation methods are consolidated on a global basis. Furthermore, the grading standard of evaluation index is constituted by the cluster analysis of the results of 6 indexes in 30 countries, combined with different countries and regions’ ecological environment and social and economic development level. Some evaluation results are presented in Table 2. Examination of the results of empirical studies on different countries and 31 provincial administrative regions of China showed that this approach provides a basis for grading the level of sustainable development; also, the evaluation results can be compared at a large spatio-temporal scale.

## 5. Conclusions

An evaluation system of sustainable development based on the Footprint Family has been proposed to analyze the spatio-temporal dynamic variation of *EF*, *CF*, *WF,* and sustainable development in China from 1990 to 2015. The conclusions are as follows:

(1). A sustainable development evaluation system comprising the indexes *EPI*, *EOI*, *EECI, WSI, CEI,* and *SDI* was described. This system can give comprehensive results regarding regional sustainable development from five aspects—ecological pressure, economic development, eco-economic coordination, climate change, and resource constraint. This new approach has extended the evaluation scope of the traditional Footprint Family, which has mainly concentrated on *EF*, *CF*, and *WF*. In addition, the ranking standard of the index in the sustainable evaluation system was given by cluster analysis of the calculated results for six indexes in 30 countries of the world.
(2). The development situation in China deteriorated from 1990 to 2015; the results showed that the *SDI* decreased from a medium level (grade 5) to a lower-medium level (grade 4). During this period, the *EPI* increased from a very low level (grade 2) to a medium level (grade 5), the *EOI* increased from an extremely low level (grade 1) to a low level (grade 3), the *EECI* remained consistently at a poor level (grade 1), the *CEI* increased from a low level (grade 3) to alower-medium level (grade 4), and the *WSI* increased from a low level (grade 3) to a medium level (grade 5).
(3). At the spatial level, the *SDI* was at the worst state (grade 1~3) in the eastern area, except Hainan, but including Chongqing, while the *SDI* was at the best state (grade 7~9) in Inner Mongolia, Qinghai, and Tibet in the western area, and in the middle state (grade 5) for the remaining regions in the majority of areas in the Western Area of China. The *EPI* was at its worst state in the eastern area, except Hainan, Hebei, and Shandong, and was in a good situation in the majority of regions including the western and the middle-areas of China except Sichuan, Chongqing, Hubei, Hunan, Guizhou, Hebei, Anhui, and Jiangxi. For the remaining regions, the *EPI*s were in the middle state. The *EOI* was worst in the majority of regions in China except Inner Mongolia, Shanxi, Xinjiang, Jilin, Hebei, and Jiangsu. The *EECI* was also in a bad situation except for Tibet, Xinjiang, Qinghai, Ningxia, Inner Mongolia, Shanxi, and Heilongjiang. The *CEI* was at its worst condition in Inner Mongolia, Shanxi, Ningxia, Jilin, Beijing, Tianjin, Hebei, Shandong, Shanghai, Jiangsu, and Zhejiang, while it was at the middle state for the remaining regions. The *WSI* was at its worst state in Beijing, Tianjin, Hebei, Shanxi, Ningxia, Shandong, Jiangsu, and Shanghai, was at a medium state in Xinjiang, Gansu, Shaanxi, Henan, Hubei, and Guangdong, and it was in a good state in the remaining regions.
(4). Empirical studies showed that the key factors that restricted sustainable development in China were ecological pressure, GHG emissions, and water stress. Energy savings, emissions reductions, and water savings are the corresponding measures that need to be implemented to achieve sustainable development in the future. The analysis also showed that the evaluation system of sustainable development based on the Footprint Family is not affected by human subjectivity, overcomes the deficiencies of existing evaluation systems, and can be applied to the evaluation of sustainable development under different spatio-temporal conditions.

## Figures and Tables

**Table 1:** The grading criteria for each indicator in the sustainable development evaluation system.

Grade	1	2	3	4	5	6	7	8	9
Token State	Extremely Low	Very Low	Low	Lower-Medium	Medium	Upper-Medium	High	Very High	Extremely High
*EPI*	<0.30	0.30~0.39	0.40~0.49	0.5~0.59	0.60~0.69	0.70~0.79	0.80~0.89	0.90~0.99	≥1.0
*EOI*	<0.20	0.20~0.29	0.30~0.39	0.40~0.49	0.50~0.59	0.60~0.69	0.70~0.79	0.80~0.89	≥0.90
*EECI*	<0.20	0.20~0.29	0.30~0.39	0.40~0.49	0.50~0.59	0.60~0.69	0.70~0.79	0.80~0.89	≥0.90
*CEI*	<0	0~0.06	0.07~0.19	0.20~0.29	0.30~0.39	0.40~0.49	0.50~0.59	0.60~0.69	≥0.70
*WSI*	<0.20	0.20~0.29	0.30~0.39	0.40~0.49	0.50~0.59	0.60~0.69	0.70~0.79	0.80~0.89	≥0.90
*SDI*	<0.20	0.20~0.29	0.30~0.39	0.40~0.49	0.50~0.59	0.60~0.69	0.70~0.79	0.80~0.89	≥0.90

Note: *EPI*: the ecological pressure index; *EOI*: the ecological occupancy index; *EECI*: the ecological economic coordination index; *CEI*: the GHG (Greenhouse Gas) emission index; *WSI*: the water resources stress index; *SDI*: the sustainable development index.

**Table 2:** The sustainability assessment results for different countries.

Countries	Grade of *EPI*	Grade of *EOI*	Grade of *EECI*	Grade of *CEI*	Grade of *WSI*	Grade of *SDI*
Japan	9	5	1	9	5	2
Korea	9	5	2	9	6	2
Netherlands	9	8	2	9	9	2
India	4	1	1	3	9	3
Germany	3	5	4	8	9	3
Iran	3	3	2	7	9	3
Italy	7	5	2	7	7	3
South Africa	3	3	3	7	9	3
China	5	3	1	4	5	4
Mexico	4	4	2	5	6	4
Thailand	5	3	1	5	5	4
United Kingdom	6	6	3	7	4	4
Poland	3	5	4	6	5	4
France	3	6	5	6	7	5
USA	1	9	9	9	2	6
Brazil	1	3	9	4	1	7
Russia	1	5	9	7	1	7
Argentina	1	3	9	5	3	7
Canada	1	8	9	9	1	7
Australia	1	8	9	9	1	7
